# [^18^F]Fluoride uptake in various bone types and soft tissues in rat

**DOI:** 10.1186/s13550-023-00969-4

**Published:** 2023-03-13

**Authors:** Nina Savisto, Tove J. Grönroos, Vesa Oikonen, Johan Rajander, Eliisa Löyttyniemi, Jörgen Bergman, Sarita Forsback, Olof Solin, Merja Haaparanta-Solin

**Affiliations:** 1 Radiopharmaceutical Chemistry Laboratory, Turku PET Centre, University of Turku, 20520 Turku, Finland; 2 PET Preclinical Imaging, Turku PET Centre, University of Turku, 20520 Turku, Finland; 3 MediCity Research Laboratories, University of Turku, 20520 Turku, Finland; 4 Department of Oncology and Radiotherapy, Turku University Hospital, 20520 Turku, Finland; 5 Turku PET Centre, University of Turku, 20520 Turku, Finland; 6 Accelerator Laboratory, Åbo Akademi University, 20520 Turku, Finland; 7 Department of Biostatistics, University of Turku, 20520 Turku, Finland; 8 Department of Chemistry, University of Turku, 20500 Turku, Finland; 9 PET Preclinical Laboratory/MediCity, University of Turku, Tykistökatu 6 A, 20520 Turku, Finland

**Keywords:** PET, [^18^F]NaF, Biodistribution, Metabolism, Bone uptake, Bone perfusion

## Abstract

**Background:**

In the development of new ^18^F-labelled tracers, it is important to assess the amount of released [^18^F]fluoride taken up in the bones of experimental animals because all ^18^F-labelled PET-tracers are prone, to lesser or higher degree, to undergo defluorination, with subsequent release of [^18^F]fluoride during scanning. However, the pharmacokinetics of [^18^F]fluoride in bones and other organs of healthy rats have not been well documented in a comprehensive manner. We aimed to study pharmacokinetics of [^18^F]NaF in rats in order to increase our understanding of the biodistribution of [^18^F]fluoride originating from defluorination of ^18^F-labelled tracers. We studied [^18^F]fluoride uptake in Sprague Dawley rat bones, including the epiphyseal parts of the tibia and radius, the mandible, ilium, lumbar vertebrae, costochondral joints, tibia, radius, and ribs, with 60-min in vivo PET/CT imaging. Kinetic parameters, K_1_, K_i_, K_i_/K_1_, and k_3_ were calculated with a three-compartment model. In addition, separate groups of male and female rats were studied with ex vivo bone and soft tissue harvesting and gamma counting over a 6-h period.

**Results:**

[^18^F]fluoride perfusion and uptake varied among the different bones. [^18^F]fluoride uptake was higher in trabecular bones, due to high perfusion and osteoblastic activity, compared to cortical bones. In soft tissues, the organ-to-blood uptake ratios increased over time in the eyes, lungs, brain, testes, and ovaries during the 6 h study period.

**Conclusion:**

Understanding the pharmacokinetics of [^18^F]fluoride in various bones and soft tissues is highly useful for assessing ^18^F-labelled radiotracers that release [^18^F]fluoride.

**Supplementary Information:**

The online version contains supplementary material available at 10.1186/s13550-023-00969-4.

## Background

Fluoride binds to sites of new bone formation, and thus, it serves as a marker of bone blood flow and osteoblastic activity [1–3]. Bone contains organic components, mainly Type 1 collagen, and inorganic components, primarily hydroxyapatite, Ca_10_(PO_4_)_6_(OH)_2_, and other salts. Fluoride traverses several steps before it reaches the hydroxyapatite crystal of bone. Then, it exchanges with the hydroxyl group in hydroxyapatite and forms fluoroapatite (Ca_10_(PO_4_)_6_F_2_) [4, 5]. This process is slow, but once fluoride has entered the bound water shell surrounding the hydroxyapatite crystals it can be considered part of the bone [6].

The use of ^18^F-labelled sodium fluoride ([^18^F]NaF) as a bone seeking tracer for imaging skeletal malignancies was described by Blau and co-workers in the 60s and 70s [6, 7]. In 1993, whole-body [^18^F]NaF imaging with positron emission tomography (PET) was introduced for clinical use [8].

The most common indications for [^18^F]NaF imaging are benign and metabolic bone diseases. These indications for the clinical use of [^18^F]NaF were exhaustively discussed in a recent EANM guidelines by Beheshti et al. [9].

In addition to the clinical uses, it is important to improve our understanding of [^18^F]fluoride behaviour in vivo to facilitate the development of new ^18^F-labelled PET tracers, which are widely used in PET studies. Most ^18^F-labelled tracers will, at some point in their metabolism, lose the ^18^F-label, which is released as [^18^F]fluoride. However, in studies where defluorination of an ^18^F-labelled compound was analysed by measuring its uptake in bone, the site on the bone that was sampled was rarely mentioned. In addition, it is important to know the potential uptake of [^18^F]fluoride in soft tissues, when considering accurate modeling of a tracer.

Knowledge of [^18^F]fluoride pharmacokinetics can improve the interpretation of clinical [^18^F]NaF PET images and increase our understanding of the biodistribution of ^18^F-labelled tracers that release [^18^F]fluoride. Hence, the purpose of this study was to determine [^18^F]fluoride uptake as a function of time in different bones and soft tissues in healthy Sprague Dawley rats.

## Methods

### ***Production of [***^***18***^***F]NaF***

No-carrier-added aqueous [^18^F]fluoride was produced according to previously described procedures [10]. Briefly, an ion-exchange resin (Sep-Pak Accell Light QMA cartridge, Waters Corporation, Milford, MA, USA) was prepared by washing with NaCl (0.7 mL, 0.9 mg/mL) in water (9.3 mL). Next, aqueous [^18^F]fluoride was drawn into a collection syringe and passed through the anion-exchange resin in the carbonate form. The trapped [^18^F]fluoride was rinsed with sterile water (10 mL) to remove contaminants and traces of irradiated water. Finally, [^18^F]NaF was eluted from the ion-exchange cartridge with a NaCl-solution (10 mL, 9 mg/mL). The [^18^F]NaF was then formulated for injection using a sterile filtration unit combined with the synthesis device. The end product was filtered through a sterile filter (Millex GP 0.22 µm, EDM Millipore Billerica, MA, USA), and placed into a sterile, pyrogen-free, vial.

### Animals

We used Harlan Sprague–Dawley rats (n = 39, 21 males and 18 females), bred and housed at the Central Animal Laboratory, University of Turku, Turku, Finland. The males were younger (55 ± 16 d) than the females (112 ± 6 d), but the body weights (male; 286 ± 8 g, and female; 260 ± 25 g) were not significantly different between the sexes. All rats were housed under standard conditions (temperature 21 °C; humidity 55 ± 5%) with lights on from 6:00 a.m. to 6 p.m. The rats had free access to standard laboratory food and tap water. All animal experiments were approved by the Animal Experiment Board of the Province of Southern Finland (ESAVI/4660/04.10.07/2016), and carried out according to ARRIVE guidelines; the United Kingdom Animals (Scientific Procedures) Act, 1986, and EU Directive 2010/63/EU for animal experiments.

### ***Biodistribution of [***^***18***^***F]fluoride***

[^18^F]NaF was injected intravenously via a tail vein into male (n = 15, 32.3 ± 6.4 MBq) and female rats (n = 18, 32.2 ± 4.5 MBq) under brief isoflurane/oxygen anaesthesia. The rats (n = 6 for each time point) were sacrificed via cardiac puncture under increased anaesthesia at 15, 30, 60, 120, 240, or 360 min after injecting the tracer. Blood, urine, and organs of interest were removed, weighed, and measured for ^18^F-radioactivity in a NaI(Tl) well counter (3 × 3-inch, Bicron, Newbury, USA). The uptake of ^18^F-radioactivity was expressed as the percentage of the injected dose per gram of tissue (% inj.dose/g) or as the organ-to-blood ratio. The binding of [^18^F]fluoride to plasma proteins was determined by ultrafiltration, as previously described [11]. We used a haematocrit value of 41% [12] for calculating the portion of [^18^F]fluoride that bound to erythrocytes.

The total radioactivity in the urine was calculated by pooling the amount of radioactivity in the bladder with the amount excreted in the urine throughout the study.

### PET/CT Imaging

Three male rats (two of them twice) (272 ± 26 g) were anesthetized with an isoflurane/oxygen gas mixture. [^18^F]NaF was intravenously injected via a tail vein (18.7 ± 2.4 MBq). Rats were scanned with an Inveon multimodality PET/CT scanner (Siemens Medical Solutions, Knoxville, TN). Transmission scans with the CT modality were first performed for anatomical reference and for correcting attenuations of PET data. Dynamic PET scans were acquired in list mode with a 350–650 keV energy window. Scans were initiated at the same time that radiotracer was injected and continued for 60 min. Sinograms were framed into 53 time frames (4 × 5 s, 28 × 10 s, 15 × 60 s, 4 × 300 s, and 2 × 600 s) and reconstructed with Fourier rebinning and a two-dimensional filtered backprojection reconstruction algorithm. Volumes of interest (VOIs) were drawn over compact (cortical) bones, cancellous (trabecular) bones, and flat bones. Compact bone samples were taken from the diaphysis of long bones (tibia and radius). Cancellous bone samples were taken from the epiphyseal regions of long bones (tibia head and radial head). In addition to bone samples, VOIs were drawn over the following soft tissues; whole brain, cardiac left ventricle, liver, kidneys, bone marrow, and bladder, with Inveon Research Workplace Image Analysis software (Siemens Medical Solutions). The image voxel size was 0.86 mm × 0.86 mm × 0.80 mm. From the VOIs, time–activity curves (TACs) were obtained. [^18^F]fluoride uptake was expressed as the percentage of the injected dose per millilitre of tissue (% inj.dose/mL).

### Kinetic modeling

For kinetic analyses, we used the whole blood TAC, measured from the left cardiac ventricle as the input function, in images where the heart was in the field of view (n = 3 rats). Fluoride is rapidly transported through the red blood cell membrane; thus, [^18^F]fluoride in whole blood is available for exchange in tissue capillaries [2, 13]. Blood TACs could be reliably determined from the image, because the ventricle was relatively large compared to the image resolution, and the activity ratio between myocardium and blood remained stable during the PET study (Additional file 1: Table S2). The net [^18^F]fluoride influx rate (K_i_) in bone was assessed with a Patlak graphical analysis. The rate constants for [^18^F]fluoride transport from blood to the extravascular compartment (K_1_) and back to the blood (k_2_), the rate constants for binding to and detaching from hydroxyapatite at the bone surface (k_3_ and k_4_), and the vascular volume fraction (V_b_) were parameters of the three-compartmental model [14–16] (Additional file 1: Fig. S1). The rate constant, K_1_, mainly represented [^18^F]fluoride perfusion. The ratio K_i_/K_1_ (in terms of the compartmental model: k_3_/(k_2_ + k_3_), was the unidirectional extraction efficiency from blood to bone minerals [17]. Detachment of fluoride-18 from bone mineral was assumed negligible during the PET study (k4 = 0).

### Statistics

The statistical analyses were performed using the SAS software, version 9.4 for Windows (SAS Institute Inc., Cary, NC, USA). The K_1_, K_i_, K_1_/K_i_ were compared for more bone in the same rat, therefore the analysis was done using repeated measures techniques, where bone is a repeated factor (hierarchical linear mixed model). While overall differences were detected between the bones, pairwise comparisons were made between the bones. Urinary excretion of radioactivity in females and males was analysed using two-way analysis of variance (ANOVA), using time and gender as explanatory variables (at each time point different rat was measured). Values are expressed as the means ± standard deviation (SD). *P* values < 0.05 (two-tailed) were considered significant.

## Results

### ***Production of [***^***18***^***F]NaF***

The radiochemical and radionuclidic purity of [^18^F]NaF exceeded 98.5% in all synthesized batches.

### ***Pharmacokinetic properties of [***^***18***^***F]fluoride***

**Fig. 1:**
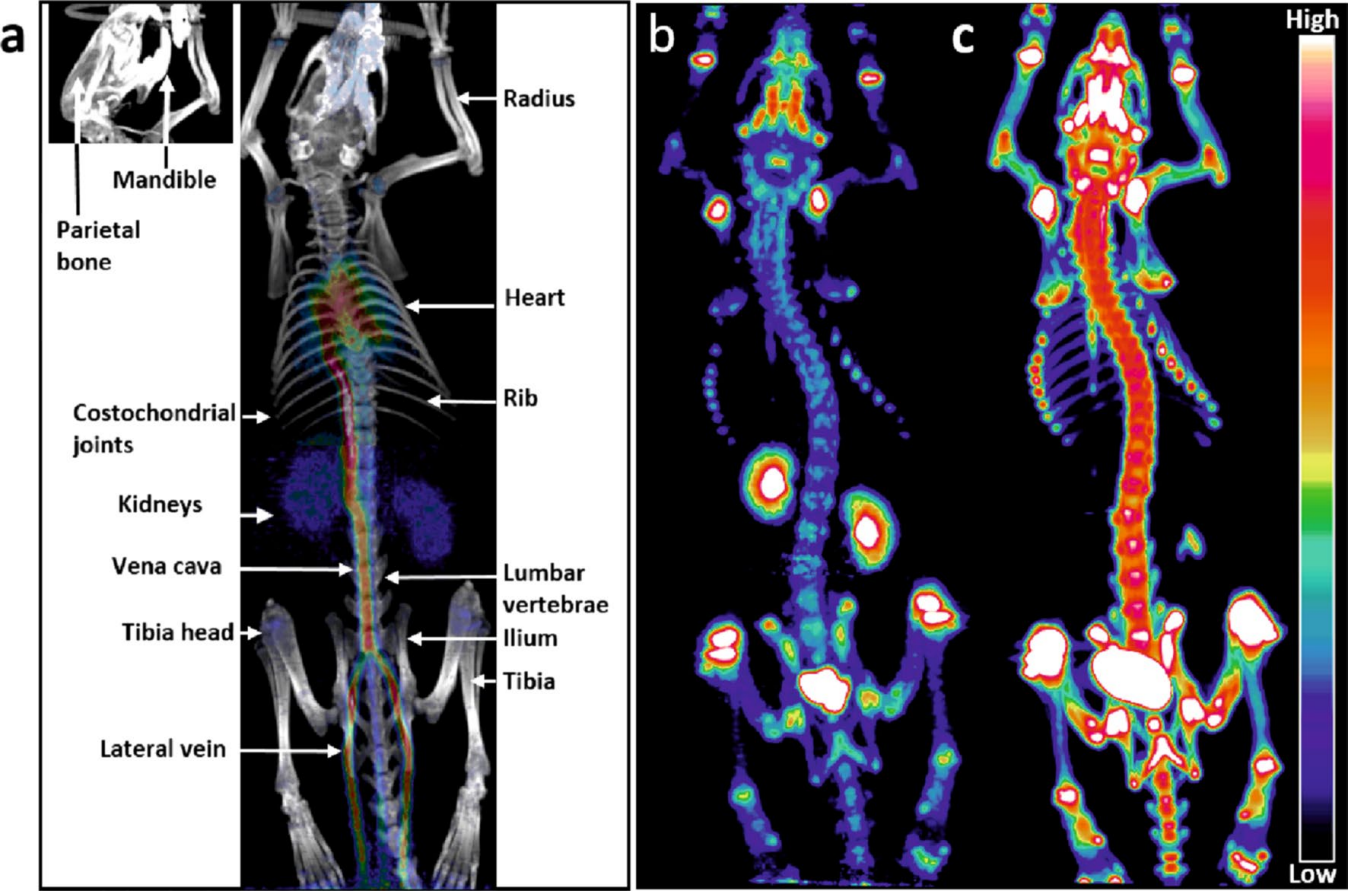
Fused anterior 3D PET/CT image acquired at 0–30 s and PET images acquired at 1–5 min and 20–50 min show the in vivo biodistribution of [^18^F]fluoride in male Sprague Dawley rats (the images present the same rat but from two different imaging sessions). **a** At 0–30 s, the highest uptakes are observed in the caudal vein, vena cava, heart, and kidneys. **b** At 1–5 min after the injection, uptake appears in the kidneys, bladder, and epiphyseal regions of long bones, mandible, and costochondral joints. **c** At 20–50 min, uptake is decreased in the kidneys but elevated in the bones and bladder

**Fig. 2:**
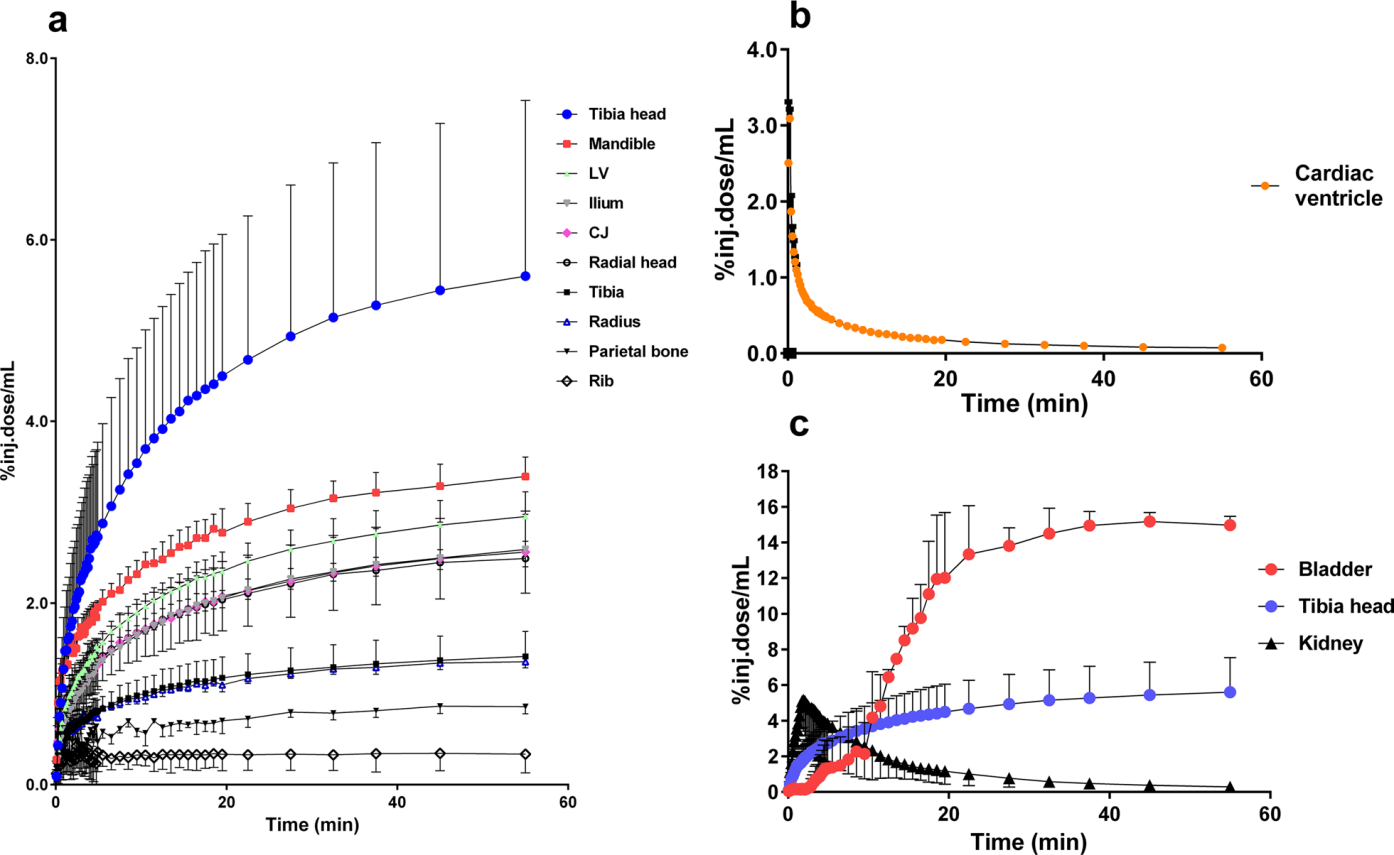
**a** Time-activity curves (TACs) obtained from VOIs of different rat bones. **b** The TAC of the left cardiac ventricle demonstrates the rapid clearance of [^18^F]fluoride from blood. **c** Uptake in kidney and bladder represent excretion of [^18^F]fluoride via the urinary tract; the TAC for the tibia shows an example of [^18^F]fluoride uptake in the diaphysis of a long bone. LV = Lumbar vertebrae, CJ = costochondral joint. Values are the mean ± SD, n = 3–5

In vivo PET imaging revealed a rapid clearance of [^18^F]fluoride from blood, based on measurements in the left cardiac ventricle. High uptake was observed in the kidneys only a few minutes after tracer injection (Figs. 1, 2b and c).

Ex vivo measurements confirmed, that [^18^F]fluoride was rapidly cleared from the blood. Furthermore, 47 ± 4% of the blood radioactivity was bound to erythrocytes. This amount remained constant over the 6-h time course of this study (Additional file 1: Fig. S3a and Table S1). The binding of [^18^F]fluoride into plasma proteins was negligible.

PET imaging revealed a high uptake of radioactivity in the epiphyseal regions of the long bones, which represented cancellous bone. The mandible, lumbar vertebrae, and parts of the pelvis also showed high uptake. Uptake was higher in the costochondral joints (*p* < 0.0005) than in other parts of the ribs. The lowest in vivo uptake was observed in compact bones, like the tibia, radius, and parietal bone (Figs. 1 and 2a).

**Fig. 3:**
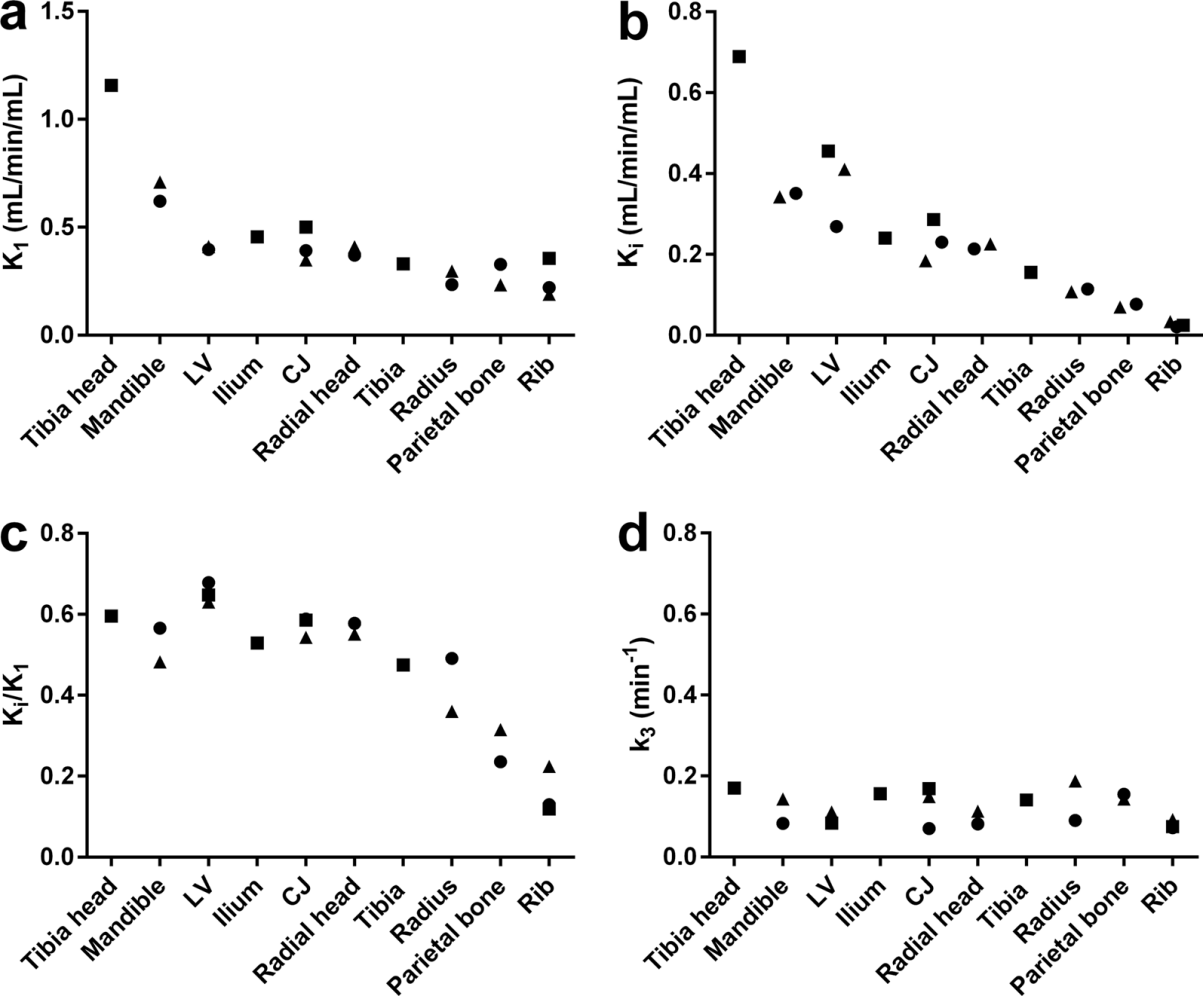
Individual kinetic values derived from a model of [^18^F]fluoride uptake for different rat bones. **a** K_1_: describes bone perfusion; **b** K_i_: reflects bone osteoblastic activity; **c** K_i_/K_1_: describes the efficiency of unidirectional extraction from blood to bone; and **d** k_3_: the rate constant from the extravascular compartment to the bone bound compartment. Values are based on measurements in 1–3 male rats. Different symbols represent different rats. LV = lumbar vertebrae, CJ = costochondral joint

The mean bone perfusion rates (K_1_) were significantly higher (*p* < 0.0001) in the tibia head and mandible (*p* < 0.001) than in the other measured bones (Fig. 3a, Additional file 1: Fig. S4a). The osteoblastic activity in bone, based on the K_i_ value, was higher in the tibia head (*p* ≤ 0.001), the mandible (*p* < 0.01), and the lumbar vertebrae (*p* ≤ 0.004), than in the tibia, radius, parietal bone, or rib (Fig. 3b, Additional file 1: Fig. S4b). The K_i_/K_1_ ratio, which described the unidirectional extraction efficiencies from blood to bone mineral were significantly (*p* ≤ 0.02) lower in the parietal bone, and rib than in the other measured bones (Fig. 3c, Additional file 1: Fig. S4c). The rate constant of transfer from the extravascular compartment to the bone bound compartment, k_3_, was similar in all measured bones (Fig. 3d).

After the 60 min scan, the ex vivo radioactivity uptake was measured in bones. The highest uptakes (% inj.dose/g) were measured in the tibia head and costochondral joints. Uptakes were similar in the mandible, tibia, rib, ilium, and parietal bone (Table 1). The [^18^F]fluoride uptake into the parietal bone was also measured at different time points after tracer injection (from 15 to 360 min). The highest value was observed at 120 min (2.5 ± 0.2 % inj.dose/g; Additional file 1: Table S1). The uptake in bone marrow varied between 0.1 and 0.3 % inj.dose/g over the 6 h study period (Additional file 1: Table S1).

**Table 1:** Uptake as % of injected dose/gram of tissue (% inj.dose/g) and the bone-to-blood ratios of ^18^F-radioactivity detected ex vivo in different bones at 60 min after [^18^F]NaF injection

Bone	Uptake (% inj.dose/g)	Bone/blood
Tibia head	13 (3)	281 (87)
Mandible	4.2 (2.9)	105 (55)
CJ	6.1 (1.9)	131 (50)
Ilium	3.4 (0.4)	71 (10)
Tibia	4.6 (2.0)	72 (23)
Rib	2.1 (0.4)	45 (7.3)
Parietal bone	2.0 (0.6)	36 (23)

Values are the means (SD); n = 6 for parietal bone and n = 3–5 for other bones

*% inj.dose/g* percent of the injected dose per gram of tissue, *CJ* costochondral joint

**Fig. 4:**
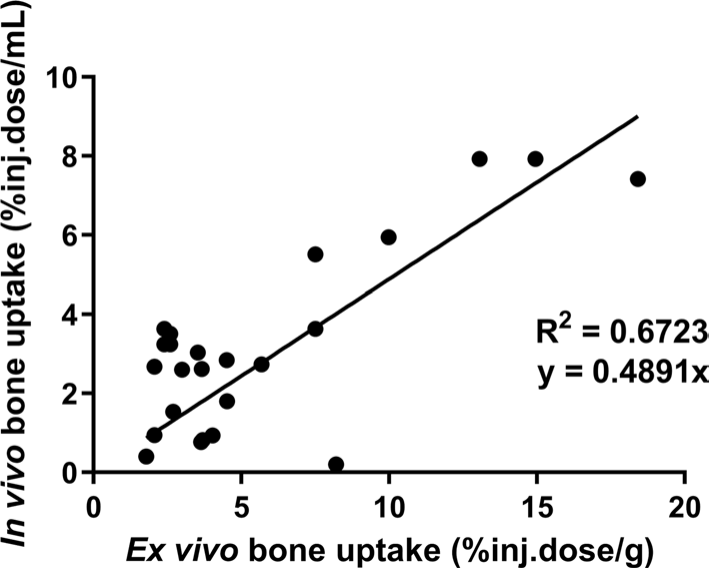
Correlation between in vivo (% inj.dose/mL) and ex vivo (% inj.dose/g) measurements of [^18^F]fluoride uptake into bone, measured at 60 min after tracer injection

Bone uptakes measured in vivo correlated well (r = 0.8199) with the ex vivo measurements (Fig. 4). The slope (0.4891) of the correlation revealed that the in vivo uptake values were about half the values measured in the ex vivo setting.

**Fig. 5:**
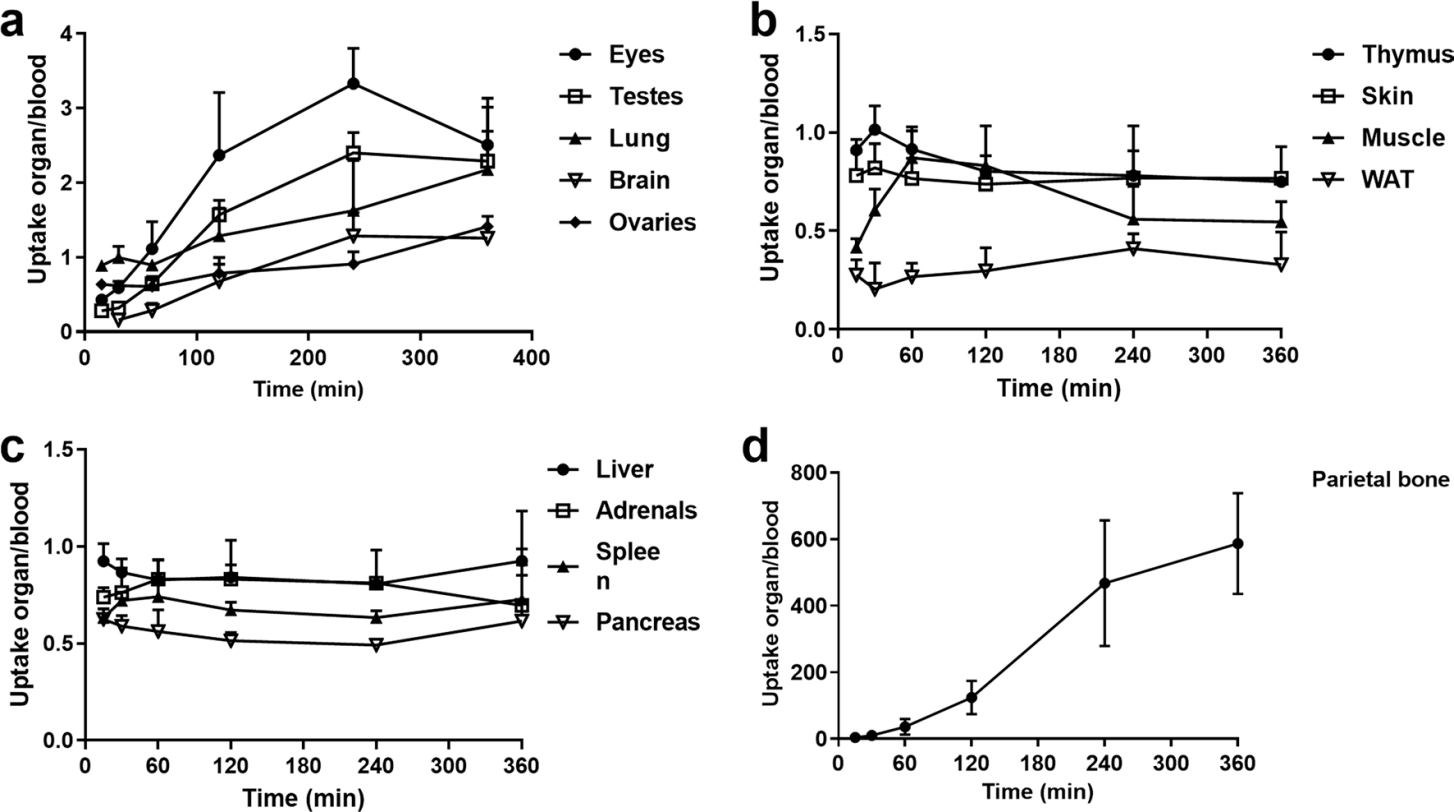
Ex vivo organ-to-blood ratios of [^18^F]fluoride at 15, 30, 60, 120, 240, and 360 min after injection. **a** Ratios increased in the eyes, testes, lung, brain, and ovaries. **b** Ratios remained unchanged over the studied 6 h in white adipose tissue (WAT), skin, muscle, and thymus, and **c** in liver, adrenals, spleen, and pancreas. **d** The parietal bone-to-blood ratio increased over the 6 h time course. Values are the mean ± SD, n = 3 for testis and ovary and n = 6/time point for other organs

In soft tissues, the organ-to-blood uptake ratios increased and plateaued over time in the eyes, testes and brain, and increased in lungs, and ovaries during the 6 h study period (Fig. 5a, Additional file 1: Table S2). In most soft tissues, the organ-to-blood ratio remained essentially constant. This finding indicated that the tracer clearance rate from blood to various organs resembled the blood reuptake rate (Fig. 5b and c, Additional file 1: Table S2). In the parietal bone, the bone-to-blood ratio increased throughout the 6 h study (Fig. 5d).

The total amount of radioactivity excreted in the urine increased slowly over the entire 6-h ex vivo measurement period, and it ultimately reached 26 ± 7% of the total injected dose. Urinary tract excretion was significantly (*p* = 0.0001) faster in female rats than in the male rats (Additional file 1: Fig. S3c). Although [^18^F]fluoride was mainly excreted via the urinary tract, we also observed increasing [^18^F]fluoride uptake in the small intestine, up to 120 min after tracer injection. After 240 min, uptake also increased in the large intestine (Additional file 1: Fig. S3b and Table S1).

## Discussion

The results of this study were relevant to both clinical and experimental settings. First, our findings on the pharmacokinetic properties of [^18^F]fluoride in different bones as a function of time may be used to adapt tracer protocols to fit different clinical applications, and when assessing the bone uptake of ^18^F-labelled tracers that release [^18^F]fluoride. Second, our findings on the behaviour of free [^18^F]fluoride in soft tissues improved our understanding of when defluorination occurs in the metabolism of ^18^F-labelled tracers; this information might have an impact on future kinetic modeling approaches.

It is well known that [^18^F]fluoride is taken up by bones; for example, the rate of [^18^F]fluoride influx into bone mineral (K_i_) was shown to be irreversible by using a Patlak graphical analysis [3]. We studied the uptake of [^18^F]fluoride into various rat bones with both in vivo PET imaging (for 60 min after tracer injection) and ex vivo gamma counting (for 360 min after tracer injection). We demonstrated a good correlation between these measurements at 60 min post injection. With both methodologies, the highest uptake was observed in the epiphyseal region of tibia. However, the uptake in the epiphyseal regions of the tibia appeared to be more related to bone perfusion (K_1_) than to bone uptake (k_3_). K_i_ accurately describes the net influx of fluoride in tissues, but cannot separate the effect of perfusion from fluoride binding to hydroxyapatite. The Patlak graphical analysis and compartmental modeling produces parametric images of less quality, but they show that the high net fluoride uptake in certain skeletal regions is due to high perfusion (K_1_), and that fluoride binding (k_3_) in these regions is within the same range as the k_3_ observed in other skeletal regions (Additional file 1: Fig. S2). Including k_4_ in the compartmental model fit led to unstable parameter estimates in most animals.

The group of bones with the second highest uptake were the mandible, lumbar vertebrae, ilium, and costochondral joints. As shown in Fig. 3 and in Additional file 1: Figs. S2 and S4, these uptakes were bone specific (k_3_). The relatively high uptake in the mandibular bone was probably due to [^18^F]fluoride accumulation in the dentine of teeth [18, 19]. The tibia, radius, parietal bone, and ribs showed the lowest uptake. Arvola et al. found similar order in ^18^F-uptake 60 min after [^18^F]NaF injection when measuring standardised uptake values (SUVs) from normal appearing human bones spine, pelvis, limbs, rib and skull [20].

Our results indicated that [^18^F]fluoride perfusion and uptake varied among the different bones. This variation must be taken into consideration when comparing, e.g., SUVs in different bone diseases in a clinical setting or when reporting the bone uptake of [^18^F]fluoride released from the ^18^F-labelled tracer. We found the highest [^18^F]fluoride uptake in cancellous bones, and we demonstrated that this uptake depended on perfusion. These results were consistent with a previous study by Eble et al., who revealed that, after long-term fluoride exposure, fluoride concentrations were higher in cancellous bones than in compact bones [21].

As shown in Figs. 1 and 2b (in vivo) and in Additional file 1: Fig. S3a (ex vivo), [^18^F]fluoride was rapidly cleared from the blood during the first few minutes after the injection. Low tracer uptake was observed in the myocardium, therefore, the blood input function for kinetic modeling of the in vivo scanned data was measured in the left cardiac ventricle. Park-Holohan et al. suggested that fluoride was rapidly transported through red blood cell membranes, and then it was available for clearance, through uptake by the bone. Therefore, they suggested that bone kinetic data for [^18^F]fluoride would be more accurately reported as whole-blood clearance, rather than plasma clearance [13].

Our finding that [^18^F]fluoride did not bind to plasma proteins was reported previously. This property is considered an advantage of [^18^F]fluoride, compared to [^99m^Tc]diphosphonate, which showed 25–70% binding to plasma proteins [2]. Due to the lower molecular weight of fluoride compared to phosphonates, [^18^F]fluoride has a higher single-passage extraction efficiency compared to phosphonates [2]. The [^18^F]fluoride uptake we measured in bone marrow was consistent with the findings of Blake et al. [2], who speculated that the uptake in red blood cells might reflect the uptake of immature erythrocytes in bone marrow.

The rapid, high kidney uptake demonstrated that [^18^F]fluoride underwent both glomerular filtration and tubular secretion. Previously, Whitford demonstrated that fluoride was rapidly cleared from the plasma, both through renal excretion and by diffusion through capillaries. That study also showed that, due to a higher glomerular filtration capacity, renal clearance of fluoride was increased at higher pH urine levels [22]. Interestingly, we found a significant difference in the amount of total excreted urine between female and male rats (Additional file 1: Fig. S3c). This might be explained by the different anatomical and hormonal features of female and male rats [23].

Organ-to-blood ^18^F-radioactivity ratios increased as a function of time in the eyes, testes, lung, brain, and ovaries. Some of the brain tissue uptake seen with any ^18^F-labelled PET tracer, with a tendency for defluorination, might actually originate from [^18^F]fluoride. This can be a source of error in kinetic PET analysis models of the brain.

One study limitation might have been the difference in age between female and male rats, but their average weights were the same. Moreover, all rats were juvenile, rather than adults (average ages: 3.7 months for females and 1.8 months for males). A previous study showed that the bone perfusion rate and vascular resistance did not differ between juvenile (2 months) and adult (6 months) rats [24]. Hence, we assumed that the age difference did not affect the [^18^F]fluoride uptake kinetics reported in this study.

Another limitation might be that, due to the limited field of view (12.7 cm) of the Inveon PET scanner, whole body scans of the rats were not possible. Hence, 2 rats were scanned twice: first from head-to-middle and then on separate day from middle-to-hind legs. Furthermore, the small size of some bones (e.g., the ribs) resulted in small VOIs; hence, partial volumes and spill-over might have affected our in vivo results. We made pairwise comparisons between the bones but no classical adjustment (like Tukey) was performed knowing the fact that these are too strict to correlated least square means. This holds particularly when we had several bone sides, resulting in many pairwise comparisons.

## Conclusion

Our study highlighted the fact that [^18^F]fluoride uptake varied for different types of bones. It also highlighted the importance in understanding the pharmacokinetics of [^18^F]fluoride in different bones and soft tissues when assessing ^18^F-labelled radiotracers, which release substantial amounts of [^18^F]fluoride. This understanding also applies to using [^18^F]NaF in diagnostics when the disease or treatment is assessed, or monitoring therapeutic interventions that target metabolic, traumatic, or neoplastic bone diseases in appropriate animal models.

## Supplementary Information

**Additional file 1: Fig. S1. **Three-compartment model for [^18^F]fluoride kinetics in bone. **Fig. S2. **The Patlak graphical analysis and the three-compartment model were applied pixel-by-pixel to the dynamic image data from one rat to produce representative images of K_i_ and model parameters K_1_ and k_3_. **Fig. S3. **Ex vivo uptake of [^18^F]fluoride at 15, 30, 60, 120, 240, and 360 min after injection. **Fig. S4**. A graphical representation of the bone statistical data for K_1_, K_i_ and K_i_/K_1_. **Table S1** and **Table S2**.

## Data Availability

The analyses of the data supporting the conclusions of this article are included within the article and the supplementary file. The raw datasets used and analysed during the current study are available from the corresponding author on reasonable request.

## Abbreviations

[^18^F]NaF: ^18^F-labelled sodium fluoride
PET: Positron emission tomography
%inj.dose/g: Injected dose per gram of tissue
VOI: Volume of interest
TAC: Time–activity curve
% inj.dose/mL: Injected dose per millilitre of tissue
AUC: Area under curve
ANOVA: Analysis of variance
SD: Standard deviation
SUV: Standardised uptake values
WAT: White adipose tissue
