# Supplementary material for: [18F]Fluoride uptake in various bone types and soft tissues in rat
Source: EJNMMI Res. 2023 Mar 13;13:21. doi: 10.1186/s13550-023-00969-4 (PMC10011276; doi:10.1186/s13550-023-00969-4)
Supplement: Supplementary file 1 — Additional file 1: Fig. S1. Three-compartment model for [18F]fluoride kinetics in bone. Fig. S2. The Patlak graphical analysis and the three-compartment model were applied pixel-by-pixel to the dynamic image data from one rat to produce representative images of Ki and model parameters K1 and k3. Fig. S3. Ex vivo uptake of [18F]fluoride at 15, 30, 60, 120, 240, and 360 min after injection. Fig. S4. A graphical representation of the bone statistical data for K1, Ki and Ki/K1. Table S1 and Table S2. [file 13550_2023_969_MOESM1_ESM.docx]

Additional file 1

[^18^F]Fluoride uptake in various bone types and soft tissues in rat

Nina Savisto, Tove J. Grönroos, Vesa Oikonen, Johan Rajander, Eliisa Löyttyniemi, Jörgen Bergman, Sarita Forsback, Olof Solin, Merja Haaparanta-Solin

**
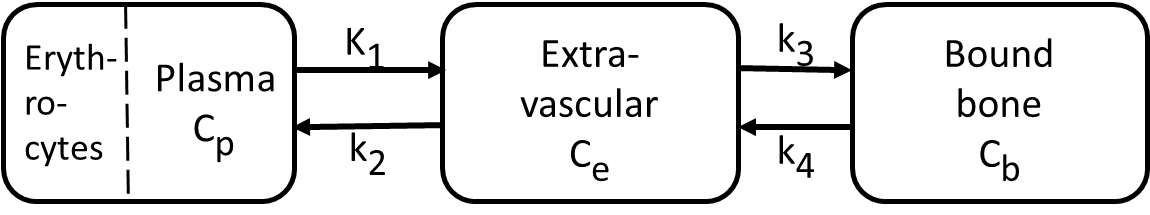
**

**FIGURE S1** Three-compartment model for [^18^F]fluoride kinetics in bone. C_p_: plasma, C_e_: extravascular, and C_b_: bound bone compartments. K_1_, k_2_, k_3_, and k_4_ are rate constants that describe transport between compartments (Hawkins et al. 1992).

**
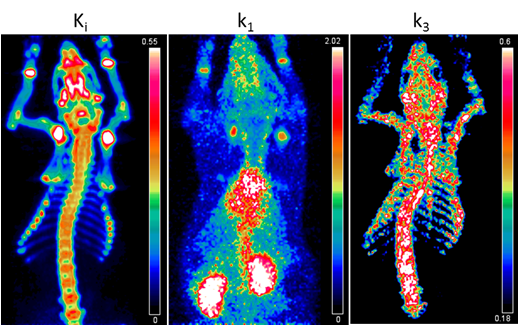
**

**FIGURE S2.** The Patlak graphical analysis and the three-compartment model were applied pixel-by-pixel to the dynamic image data from one rat to produce representative images of K_i_ and model parameters K_1_ and k_3_. In terms of the compartment model, K_i_ equals K_1_*k_3_/(k_2_+k_3_). K_i_ accurately describes the net influx of fluoride in tissues, but cannot separate the effect of perfusion from fluoride binding to hydroxyapatite. Compartmental modeling, on the other hand, produces parametric images of less quality, but they could distinguish perfusion from binding. They show that the high net fluoride uptake in certain skeletal regions is due to high perfusion (K_1_), and that fluoride binding (k_3_) in these regions is within the same range as the k_3_ observed in other skeletal regions.

**
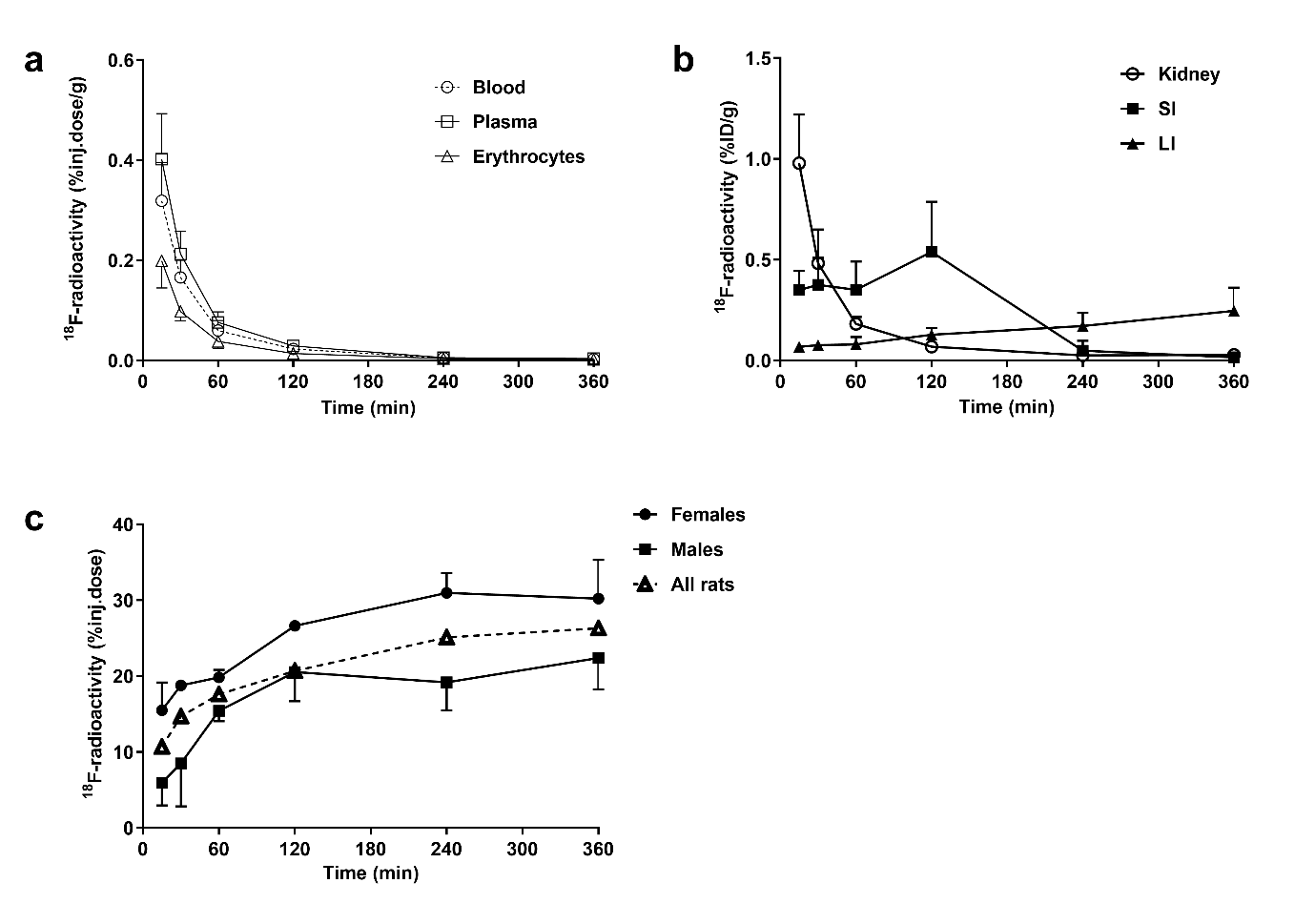
**

**FIGURE S3**. *Ex vivo* uptake (% of inj. dose/g) of [^18^F]fluoride at 15, 30, 60, 120, 240, and 360 min (n = 6/time point) after injection. (**A**) Uptake in blood, plasma, and erythrocytes; (**B**) uptake in the excretory organs: kidney, small intestine (SI), and large intestine (LI). (**C**) The total amount of [^18^F]fluoride excreted in the urine (% of injected dose), presented separately for males (n = 3) and females (n = 3). Results are averages ± SD at each time point.


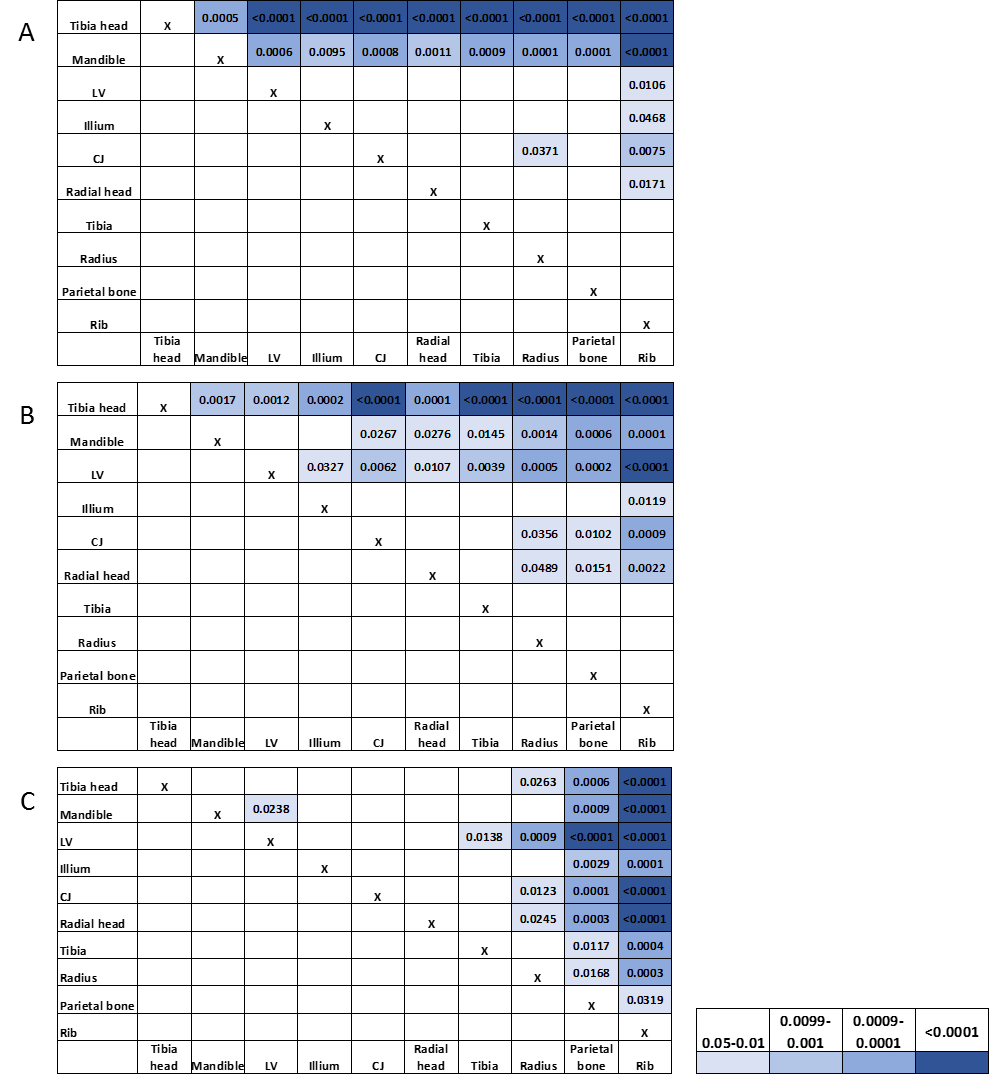


**FIGURE S4**. A graphical representation of the bone statistical data for (**A**) K_1_, (**B**) K_i_ and (**C**) K_i_/K_1_ where the individual p-values are represented also as colors. The confidence intervals are presented in colour codes. LV = lumbar vertebrae, CJ = costochondral joint

**Table S1.** Uptake of ^18^F radioactivity in organs as % of injected dose/gram of tissue of interest in Sprague Dawley rats at various time points after injection of [^18^F]NaF

| **Organ** | **15 min** | **30 min** | **60 min** | **120 min** | **240 min** | **360 min** |
| --- | --- | --- | --- | --- | --- | --- |
| **Blood** | 0.34 (0.07) | 0.17 (0.03) | 0.07 (0.02) | 0.022 (0.008) | 0.005 (0.002) | 0.004 (0.001) |
| **Plasma** | 0.42 (0.08) | 0.22 (0.04) | 0.08 (0.03) | 0.03 (0.01) | 0.006 (0.002) | 0.004 (0.001) |
| **Erythrocytes** | 0.20 (0.05) | 0.10 (0.02) | 0.04 (0.01) | 0.014 (0.005) | 0.004 (0.001) | 0.002 (0.001) |
| **PB** | 1.4 (0.3) | 1.8 (0.2) | 2.0 (0.6) | 2.5 (0.2) | 2.3 (0.4) | 2.0 (0.2) |
| **BM** | 0.29 (0.16) | 0.10 (0.06) | 0.22 (0.18) | 0.27 (0.28) | 0.26 (0.23) | 0.19 (0.15) |
| **Brain** | 0.025 (0.005) | 0.028 (0.008) | 0.018 (0.006) | 0.014 (0.003) | 0.007 (0.002) | 0.004 (0.001) |
| **Eyes** | 0.14 (0.02) | 0.10 (0.02) | 0.07 (0.02) | 0.05 (0.01) | 0.018 (0.006) | 0.009 (0.003) |
| **SG** | 0.22 (0.04) | 0.12 (0.03) | 0.04 (0.01) | 0.015 (0.006) | 0.004 (0.001) | 0.002 (0.001) |
| **Thyroid** | 0.22 (0.16) | 0.10 (0.04) | 0.05 (0.01) | 0.04 (0.02) | 0.02 (0.02) | 0.06 (0.11) |
| **Thymus** | 0.30 (0.06) | 0.18 (0.04) | 0.06 (0.02) | 0.018 (0.007) | 0.004 (0.001) | 0.003 (0.001) |
| **Heart** | 0.17 (0.04) | 0.09 (0.02) | 0.035 (0.009) | 0.014 (0.004) | 0.005 (0.002) | 0.004 (0.002) |
| **Lung** | 0.30 (0.08) | 0.18 (0.06) | 0.06 (0.02) | 0.03 (0.02) | 0.009 (0.006) | 0.008 (0.005) |
| **Liver** | 0.31 (0.04) | 0.15 (0.03) | 0.05 (0.01) | 0.019 (0.006) | 0.004 (0.001) | 0.003 (0.001) |
| **Spleen** | 0.21 (0.04) | 0.12 (0.02) | 0.05 (0.01) | 0.015 (0.006) | 0.003 (0.001) | 0.003 (0.001) |
| **Pancreas** | 0.21 (0.03) | 0.10 (0.02) | 0.036 (0.007) | 0.011 (0.004) | 0.003 (0.001) | 0.002 (0.001) |
| **Kidney** | 0.98 (0.24) | 0.48 (0.17) | 0.18 (0.04) | 0.07 (0.02) | 0.03 (0.02) | 0.03 (0.03) |
| **Adrenals** | 0.25 (0.06) | 0.13 (0.03) | 0.05 (0.01) | 0.018 (0.006) | 0.004 (0.001) | 0.002 (0.001) |
| **Muscle** | 0.14 (0.02) | 0.10 (0.02) | 0.06 (0.01) | 0.02 (0.01) | 0.003 (0.002) | 0.002 (0.001) |
| **WAT** | 0.09 (0.04) | 0.04 (0.01) | 0.017 (0.006) | 0.007 (0.005) | 0.003 (0.001) | 0.001 (0.001) |
| **Skin** | 0.26 (0.01) | 0.14 (0.04) | 0.05 (0.01) | 0.017 (0.006) | 0.004 (0.001) | 0.003 (0.001) |
| **Testis*** | 0.079 (0.004) | 0.056 (0.015) | 0.032 (0.005) | 0.022 (0.006) | 0.010 (0.001) | 0.006 (0.002) |
| **Uterus*** | 0.28 (0.03) | 0.04 (0.02) | 0.009 (0.008) | 0.007 (0.004) | 0.008 (0.004) | 0.007 (0.004) |
| **Ovary*** | 0.25 (0.03) | 0.11 (0.02) | 0.049 (0.003) | 0.022 (0.005) | 0.006 (0.001) | 0.006 (0.005) |
| **Stomach** | 0.03 (0.02) | 0.015 (0.009) | 0.013 (0.006) | 0.02 (0.03) | 0.007 (0.005) | 0.01 (0.02) |
| **SI cont** | 0.35 (0.10) | 0.38 (0.13) | 0.35 (0.14) | 0.54 (0.25) | 0.05 (0.05) | 0.016 (0.007) |
| **LI cont** | 0.07 (0.02) | 0.08 (0.03) | 0.08 (0.04) | 0.13 (0.03) | 0.17 (0.07) | 0.25 (0.11) |
| **Urine** | 51 (65) | 77 (92) | 35 (24) | 39 (34) | 21 (15) | 14 (6) |

Values are the means (SD); n = 6/time point, except when indicated; *testes, ovaries, and uterus included n=3/time point. PB = parietal bone, BM = bone marrow, SG = salivary gland, WAT = white adipose tissue, SI = small intestine, LI = large intestine

**Table S2.** Organ-to-blood uptake ratios in organs of interest in Sprague Dawley rats at various time points after injection of [^18^F]NaF

| **Organ** | **15 min** | **30 min** | **60 min** | **120 min** | **240 min** | **360 min** |
| --- | --- | --- | --- | --- | --- | --- |
| **PB** | 4.5 (1.8) | 10.6 (1.5) | 36 (23) | 124 (51) | 468 (189) | 587 (152) |
| **BM** | 0.83 (0.42) | 0.60 (0.39) | 2.8 (2.2) | 9.9 (9.3) | 45 (36) | 48 (36) |
| **Brain** | 0.08 (0.01) | 0.16 (0.02) | 0.29 (0.09) | 0.68 (0.23) | 1.3 (0.3) | 1.7 (0.3) |
| **Eye** | 0.43 (0.05) | 0.59 (0.09) | 1.1 (0.4) | 2.4 (0.8) | 3.3 (0.5) | 2.5 (0.6) |
| **SG** | 0.66 (0.07) | 0.71 (0.06) | 0.65 (0.05) | 0.67 (0.06) | 0.67 (0.04) | 0.68 (0.08) |
| **Thyroid** | 0.63 (0.33) | 0.60 (0.16) | 0.74 (0.51) | 2.2 (1.7) | 4.7 (5.0) | 3.2 (3.5) |
| **Thymus** | 0.91 (0.05) | 1.0 (0.12) | 0.92 (0.11) | 0.80 (0.08) | 0.78 (0.13) | 0.75 (0.02) |
| **Heart** | 0.51 (0.03) | 0.52 (0.04) | 0.53 (0.04) | 0.66 (0.16) | 0.84 (0.17) | 1.1 (0.5) |
| **Lung** | 0.89 (0.05) | 0.10 (0.15) | 0.90 (0.05) | 1.3 (0.4) | 1.6 (0.7) | 2.2 (0.8) |
| **Liver** | 0.92 (0.09) | 0.87 (0.07) | 0.83 (0.10) | 0.84 (0.07) | 0.81 (0.02) | 0.93 (0.26) |
| **Spleen** | 0.63 (0.05) | 0.72 (0.04) | 0.74 (0.08) | 0.67 (0.04) | 0.63 (0.04) | 0.73 (0.18) |
| **Pancreas** | 0.63 (0.05) | 0.59 (0.05) | 0.56 (0.11) | 0.51 (0.04) | 0.49 (0.02) | 0.62 (0.24) |
| **Kidney** | 2.9 (0.3) | 2.7 (0.5) | 2.9 (0.9) | 3.3 (1.2) | 4.5 (1.7) | 7.5 (5.9) |
| **Adrenals** | 0.74 (0.05) | 0.76 (0.13) | 0.83 (0.10) | 0.83 (0.20) | 0.81 (0.17) | 0.70 (0.29) |
| **Muscle** | 0.42 (0.04) | 0.61 (0.11) | 0.87 (0.14) | 0.83 (0.20) | 0.56 (0.17) | 0.55 (0.10) |
| **WAT** | 0.27 (0.08) | 0.21 (0.13) | 0.27 (0.07) | 0.30 (0.12) | 0.41 (0.08) | 0.33 (0.17) |
| **Skin** | 0.78 (0.12) | 0.82 (0.12) | 0.77 (0.10) | 0.74 (0.05) | 0.77 (0.26) | 0.77 (0.16) |
| **Testis*** | 0.28 (0.01) | 0.32 (0.02) | 0.65 (0.10) | 1.6 (0.2) | 2.4 (0.3) | 2.3 (0.4) |
| **Ovary*** | 0.63 (0.04) | 0.62 (0.07) | 0.61 (0.09) | 0.79 (0.21) | 0.91 (0.17) | 1.4 (1.04) |

Values are the mean (SD); n = 6/time point, except when indicated; *testes and ovaries included n=3/time point. PB = parietal bone, BM = bone marrow, SG = salivary gland, WAT = white adipose tissue

**REFERENCES**

1. Hawkins RA, Choi Y, Huang SC et al. Evaluation of the skeletal kinetics of fluorine-18-fluoride ion with PET. *J Nucl Med*. 1992;33:633-642.
